# Body composition in nonobese individuals according to vitamin D level

**DOI:** 10.1371/journal.pone.0241858

**Published:** 2020-11-09

**Authors:** Thaísa Hoffmann Jonasson, Tatiana Munhoz da Rocha Lemos Costa, Ricardo Rasmussen Petterle, Carolina Aguiar Moreira, Victória Zeghbi Cochenski Borba

**Affiliations:** 1 Serviço de Endocrinologia e Metabologia, Hospital de Clínicas da Universidade Federal do Paraná, Curitiba, Paraná, Brazil; 2 Universidade Federal do Paraná, Setor de Ciências da Saúde, Curitiba, Paraná, Brazil; San Raffaele Roma Open University, ITALY

## Abstract

Obesity is associated with lower 25-hydroxyvitamin D (25OHD) levels, but the association between 25OHD deficiency and specific body composition (BC) patterns remains unclear. The aim of this study was to analyze the correlation between 25OHD levels and BC in a population of healthy, nonobese individuals. Cross-sectional, observational study including a convenience sample of community-dwelling healthy individuals aged ≥18 years who responded to a study advertisement and were randomly selected. The participants filled out a questionnaire and had fasting blood drawn and anthropometric indices taken. Dual-energy x-ray absorptiometry was performed for BC analysis (fat and lean body mass). The subjects were divided according to 25OHD levels into three groups: I (≤20 ng/mL, vitamin D deficient), II (>20 and <30 ng/mL, vitamin D insufficient), and III (≥30 ng/mL, vitamin D sufficient). Of 299 individuals selected, 51 were excluded, yielding a final sample of 248 (128 women) who had serum 25OHD levels measured. Women presented higher 25OHD levels than men (27.8±12.0 ng/mL and 24.8±11.3 ng/mL, respectively; p = 0.03). Including both sexes, Group I had greater body mass index (BMI; 26.6±2.5 kg/m^2^) and waist circumference (WC; 91.8.8±9.1 cm) compared with the other groups. Group I also had 75.7% and 65.3% of abnormal BMI and WC values, respectively, (p<0.05 for both) and a higher percentage of trunk and android fat confirmed by multivariate analysis. No differences in BC were observed in individuals with insufficient versus sufficient 25OHD levels. Individuals with lower 25OHD levels had increased fat in the android region and trunk. This study confirms the association of lower 25OHD levels with greater BMI and WC and increased deposition of fat in body compartments, which, even in nonobese individuals, are commonly associated with increased metabolic risk.

## Introduction

Vitamin D, originally known for its effects on bone metabolism [1], has been recently associated with several chronic diseases [2], including obesity, metabolic syndrome, hypertension, and type 2 diabetes mellitus [3]. An inverse relationship has been shown between vitamin D levels, hypertension, and fasting hyperglycemia in adolescents, along with a strong association of low vitamin D levels with overweight and visceral fat [4].

The World Health Organization (WHO) considers obesity the greatest current threat to public health [5]. In 2016, more than 1.9 billion adults were overweight, of whom over 650 million were obese [5]. Visceral fat, in particular, has been the subject of numerous studies and is considered more detrimental to the overall health, since it increases the risk of several diseases [6] and is an established cardiovascular risk factor [7]. Recently, Stefan published a review showing the importance of visceral fat mass as a determinant of cardiometabolic diseases and type 2 diabetes [8]. However, the multifactorial etiology and metabolic consequences of obesity hinder the establishment of a causal relationship between this condition and multiple coincidental factors. Additionally, lower vitamin D concentrations have been associated with greater BMI in a meta-analysis [9] and reported in obese compared with nonobese individuals [10]. Enlarged adipocytes seem to function as a reservoir of vitamin D, leading to sequestration of this fat-soluble vitamin [11]. In contrast, some researchers believe that vitamin D deficiency may contribute to worsening obesity. Adipocytes have receptors for vitamin D, and the active form of the vitamin (1,25 dihydroxyvitamin D) can lead to lipolysis, regulate adipocyte apoptosis, and reduce fat mass [11].

Observational studies have identified that obesity is associated with vitamin D deficiency [12, 13], although no consistent evidence has pointed out to a causal relationship between these events [14]. A meta-analysis of 23 studies has shown that obesity is associated with vitamin D deficiency across various age groups [9]. Among adults, vitamin D deficiency may predispose to an increased risk of chronic conditions, including diabetes, cardiovascular diseases, different types of cancer, and excess weight [15].

Vitamin D deficiency may also affect body composition. A cross-sectional analysis including 271 healthy community-dwelling elderly individuals has reported that lower vitamin D levels are associated with increased fat mass and visceral adipose tissue [16]. Other studies have confirmed the relationship between lower vitamin D levels and total and android body fat, waist circumference (WC), and waist-to-hip ratio [17, 18]. The opposite has been observed regarding lean mass, indicating that vitamin D may be positively associated with increased lean mass, physical performance, and muscle strength [19–21].

Vitamin D status is defined by levels of 25-hydroxyvitamin D (25OHD), which is considered the reservoir of vitamin D in the body [21, 22]. Normal 25OHD values adopted by most medical societies have been conceived based on population analyses and the physiological relationship between vitamin D and bone tissue [22, 23]. Available studies on 25OHD levels and body composition have included overweight and obese individuals, while data in healthy, nonobese individuals with a wide age range are lacking. Therefore, information regarding the relationship of body composition with vitamin D deficiency and 25OHD levels in nonobese individuals is still lacking.

Based on these considerations, the objective of this study was to analyze the correlation of 25OHD levels with body composition and anthropometric data in healthy, nonobese individuals.

## Methodology

This was an observational, cross-sectional study including healthy, community-dwelling individuals. The Ethics Committee on Human Research of *Hospital de Clínicas* at *Universidade Federal do Paraná* approved the study (CAAE number 16596713.7.0000.0096). The participants were selected from an advertisement inviting the public to participate in the study and were included by convenience in the cohort between February 2016 and March 2017. The inclusion criteria were men and women between the ages of 18 and 90 years, apparently healthy, with a body mass index (BMI) between 18.5 and 29.9 kg/m^2^ and without physical disabilities or need of walking devices. The exclusion criteria were professional or nonprofessional athletes, smoking, alcohol or drug abuse, obesity, decompensated acute or chronic illnesses, or use of medications such as corticosteroids, sex hormone replacement therapy, caffeine, or any other drug or diseases known to modify body composition.

After signing an informed consent, the participants filled out a questionnaire about demographic and clinical (comorbidities and medication in use) data and calcium intake calculated from dairy food on daily diet (classified by age according to the Institute of Medicine [24]), along with another questionnaire about physical activity (International Physical Activity Questionnaire [IPAQ] short form). All participants had anthropometric measurements taken by the same investigator and fasting blood collected by a trained technician.

According to the results of the IPAQ short form [25], the participants were classified as sedentary (physical activity performed for less than 10 continuous minutes/week), insufficiently active (physical activity for at least 10 continuous minutes/week), or active (vigorous physical activity for at least 20 minutes per session, at least 3 days/week, or moderate activity or walking for 30 minutes / > 5 days/week, or any combination of activities for > 150 minutes/week for > 5 days/week) [26].

Weight (kg) was measured using a digital electronic scale with a maximum capacity of 200 kg and accuracy of 50 g with the participants wearing light clothing. Height (m) was measured with the participants’ back straight, heels together, and arms extended alongside the body, while BMI was calculated as weight (kg)/squared height (m^2^) [27]. Following the WHO recommendations, BMI values between 18.5 and 24.9 kg/m^2^ were considered normal, while values between 25 and 29.9 kg/m^2^ were considered overweight [28]. According to the Consensus Statement of the IAS and ICCR Working Group on Visceral Obesity [29], WC was measured after expiration using an inelastic tape at the midpoint between the iliac crest and the last rib with the subject standing.

Laboratory tests included serum calcium level evaluated by Arsenazo III (interassay variation 5%, normal values [NV] between 8.6 and 10.3 mg/dL), fasting plasma glucose measured using the hexokinase/glucose-6-phosphate-dehydrogenase method (NV <100 mg/dL), 25OHD measured by chemiluminescence (LIAISON 25 OH Vitamin D Total Assay, DiaSorin S.p.A., Saluggia, Italy; interassay variation 20%, sensitivity 99.5%, specificity 100%). Following the Endocrine Society Clinical Practice Guideline, the subjects were divided into three groups of vitamin D status according to 25OHD levels, namely, vitamin D deficient (< 20 ng/mL; Group I [GI]), vitamin D insufficient (≥ 20 and < 30 ng/mL; Group II [GII]), and vitamin D sufficient (≥ 30 ng/mL; Group III [GIII]) [22]. Analyses were also performed using serum 25OHD levels as a continuous variable.

All participants underwent evaluation with total body dual-energy X-ray absorptiometry (DXA) scanning equipped the enCORE 2002 software (Lunar Prodigy Advance, GE Medical Systems Lunar, Madison, WI, USA; standard error 1.6%). Measurements of fat and lean mass of total body, upper limbs, lower limbs, trunk, and abdomen were analyzed. Fat percentages were analyzed separately for the limbs (legs and arms individually), trunk, and android and gynoid regions [30].

### Statistical analysis

All statistical analyses were performed using the R Core Team (2018), version 3.4.4 (R Foundation for Statistical Computing, Vienna, Austria) [31]. The results were described as median (minimum and maximum), mean, and standard deviation values for quantitative variables, or as frequency and percentage values for categorical variables. Pearson’s correlation coefficient was used to estimate the correlation between two quantitative variables. One-way analysis of variance (ANOVA) followed by a *post hoc* least significant difference (LSD) test was used to compare groups defined by vitamin D levels (deficient, insufficient, and sufficient) concerning quantitative variables. Qualitative variables were compared using the chi-square test. The condition of normality of the variables was evaluated using the Kolmogorov-Smirnov test, and variables without normal distribution were submitted to logarithmic transformation. Multivariate analysis was performed using the Tweedie regression model, with independent variables chosen by the stepwise method and results shown as relative risks (RRs) with 95% confidence intervals (CIs). P values < 0.05 indicated statistical significance.

## Results

In total, 299 individuals were screened and 51 were excluded for different reasons (obesity 15.7%, chronic disease 19.6%, athletes 5.9%, use of alcohol or smoking 39.2%, use of corticosteroids 5.9%, and hormone replacement therapy 13.7%). Finally, 248 individuals had blood samples collected and were included in the study (128 [51.6%] women). The mean age of the final cohort was 46.06 ± 17.79 years (men, 45.08 ± 18.23 years; women, 47.05 ± 17.19 years). A total of 66 (44%) women were menopausal.

Overall, the median 25OHD concentration was 24.7 ng/mL (5.6–84.7 ng/mL). The groups divided according to 25OHD levels were comparable in age, race, number of comorbidities, and serum calcium and glucose levels. The mean serum calcium level in each 25OHD group was within the normal range but was lower in GI compared with GIII (p = 0.012) (Table 1).

**Table 1:** Demographic data, laboratory results, and comorbidities according to vitamin D level.

	GROUP I (N = 75)	GROUP II (N = 105)	GROUP III (N = 68)	P VALUE*
**AGE, YEARS (MEAN ± SD)**	49.4 ± 17.6	49.4 ± 19.2	52.6 ± 19.7	0.484
**SEX**				
**WOMEN (%)**	29 (39.2%)	58 (54.7%)	41 (60.3%)	0.003
**MEN (%)**	46 (60.8%)	47 (45.3%)	27 (39.7%)	0.003
**RACE**				
**WHITE**	70 (93.3%)	101 (96.2%)	64 (94.1%)	1.0
**NON-WHITE**	5 (6.7%)	4 (3.8%)	4 (5.9%)	1.0
**COMORBIDITIES**				
**HYPERTENSION (%)**	10 (13.5%)	24 (22.6%)	16 (23.5%)	0.233
**DM (%)**	2 (2.7%)	8 (7.5%)	6 (8.8%)	0.277
**DYSLIPIDEMIA (%)**	8 (10.8%)	24(22.6%)	13 (19.1%)	0.125
**LABORATORY**				
**SERUM 25OHD (NG/ML)**	15.4 ± 3.6	24.7 ± 2.7	40.8 ± 11.8	p < 0.001
**SERUM CALCIUM (MG/DL)**	9.6 ± 0.5	9.4 ± 0.5	9.4 ± 0.5	p = 0.393
**FASTING PLASMA GLUCOSE (MG/DL)**	90.8 ± 17.4	89.2 ± 20.2	89.1 ± 17.7	p = 0.712

*Analysis of variance (ANOVA), p values < 0.05 were considered significant. Abbreviations: SD = standard deviation; DM = diabetes mellitus; % = percentage; n = number. Group I = vitamin D deficient (25OHD < 20 ng/mL); Group II = vitamin D deficient insufficient (25OHD ≥ 20 ng/mL and < 30 ng/mL); Group III = vitamin D sufficient (25OHD ≥ 30 ng/mL) [10].

The median calcium intake was 600 mg/day (0–2500 mg/day) in the overall cohort and 535.3 mg/day (0–1600 mg/day) in GI, 644 mg/day (0–1860 mg/day) mg in GII, and 610 mg/day (0–1600 mg/day) in GIII. In all, 32.1% of the individuals had adequate calcium intake, including 44.8% of the men and 55.2% of the women (p = 0.265).

**Fig 1:**
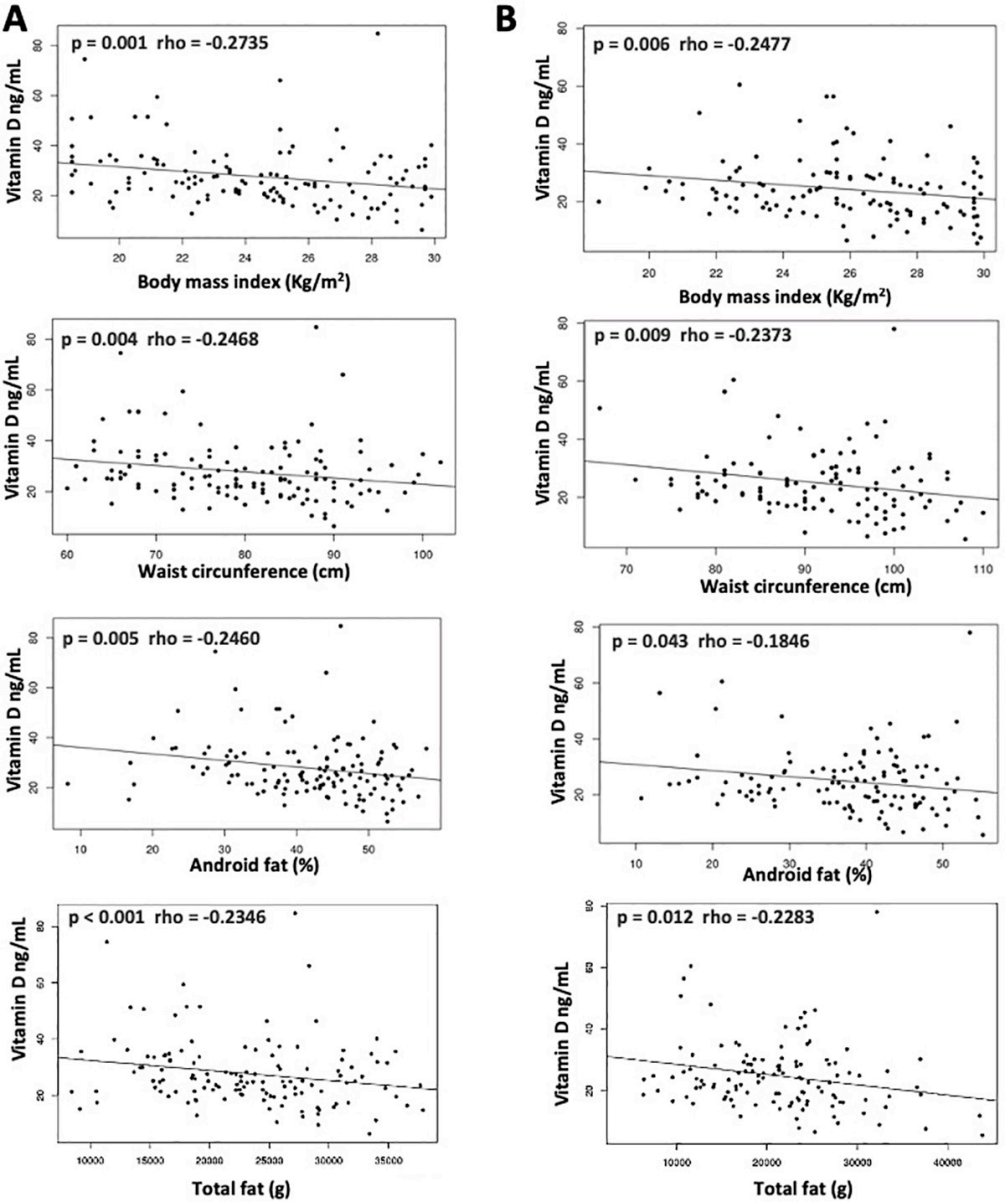
Correlation between vitamin D levels with body mass index (BMI), waist circumference, android fat, and total fat in women (A) and men (B).

Women, who comprised 60.3% of the participants in GIII, had higher 25OHD levels than men (27.8 ± 12.0 ng/mL versus 24.8 ± 11.3 ng/mL, respectively, p = 0.03). The three most frequent comorbidities were hypertension, diabetes mellitus, and dyslipidemia, all of which had similar distributions across men and women. Table 1 presents a comparison of demographic, laboratory, and comorbidity data between the groups. Values of BMI and WC were greater in GI compared with the other groups; this difference remained when men and women were analyzed separately (Table 2). Indeed, compared with the other groups, GI had a higher percentage of overweight patients with increased WC. Values of BMI and WC correlated inversely with levels of vitamin D in men and women (Fig 1).

**Table 2:** Anthropometric data according to vitamin D group.

VITAMIN D GROUPS				
**TOTAL**	I	II	III	P
**BMI (KG/M^2^)**	26.6 ± 2.5	24.5 ± 3.0	24.4 ± 3.7	^a^ 0.009
				**^b^ 0.002**
				**^c^ 0.409**
**BMI ≥ 25KG/M^2^**	27.8 ± 1.4 (75.7%)	27.4 ± 1.5 (40.6%)	27.2 ± 2.2 (51.5%)	p < 0.005
**WC (CM)**	91.3 ± 9.3	83.5 ± 10.4	83.1 ± 12.2	^a^ 0.016
				**^b^ 0.004**
				**^c^ 0.449**
**ABNORMAL WC**	67.1%	39.6%	41.2%	p < 0.005
**WOMEN**				
**BMI (KG/M^2^)**	26.0 ± 2.8	24.0 ± 2.9	23.5 ± 3.8	^a^ 0.009
				**^b^ 0.002**
				**^c^ 0.409**
**BMI ≥ 25KG/M^2^**	27.4 ± 1.5 (72.4%)	27.2 ± 1.6 (32.8%)	27.6 ± 1.8 (41.5%)	p < 0.005
**WC (CM)**	84.7 ± 7.7	79.2 ± 9.6	77.7 ± 11.1	^a^ 0.016
				**^b^ 0.004**
				**^c^ 0.449**
**WC ≥ 80CM**	75.9%	48.3%	39.0%	p < 0.005
**MEN**				
**BMI (KG/M^2^)**	26.9 ± 2.3	25.1 ± 2.9	25.7 ± 2.9	^a^ 0.001
				**^b^ 0.066**
				**^c^ 0.342**
**BMI ≥ 25KG/M^2^**	27.9 ± 1.4 (77.8%)	27.4 ± 1.5 (50.0%)	26.9 ± 1.8 (66.7%)	p < 0.005
**WC (CM)**	95.6 ± 7.5	88.7 ± 8.9	91.3 ± 8.8	^a^ < 0.001
				**^b^ 0.036**
				**^c^ 0.202**
**WC ≥ 94CM**	61.4%	29.2%	44.4%	p < 0.005

Abbreviations: BMI = body mass index; WC = waist circumference. Abnormal WC was defined as WC ≥ 80 cm in women and ≥ 94 cm in men. The results are described as mean ± standard deviation or frequency (percentage) values. Comparisons between groups

^a^ I versus II

^b^ I versus III

^c^ II versus III. Analysis of variance (quantitative variables) or chi-square test (categorical variables), p values < 0.05 were considered significant.

### Body composition

The analysis of body composition showed that individuals with lower 25OHD levels had a higher percentage of android and trunk fat, corroborating the findings of BMI and WC. Fat distribution in the limbs was not different across 25OHD groups, except for a higher % fat in the legs among men. There was a weak inverse correlation between vitamin D levels and all parameters of fat both in men and women (Fig 1). No correlation was observed between 25OHD levels and lean mass. Compared with the other groups, GI had a higher percentage of fat in the trunk and android region. This result remained when the analysis was restricted to women, while men showed a difference in percentage of fat only between GI and GII (Table 3). Calcium levels correlated inversely with the percentages of android (r = -0.14, p = 0.032) and total (r = -0.15, p = 0.019) fat.

**Table 3:** Body fat distribution between groups of vitamin D levels.

	Vitamin D groups (mean ± SD)			P
	I	II	III	
**Arms (% fat)**				
All	27.5 ± 10.3	27.7 ± 11.3	28.9 ± 10.9	0.705
Women	35.7 ± 8.5	35.2 ± 8.1	34.1 ± 9.3	0.724
Men	22.3 ± 7.5	18.6 ± 7.4	21.0 ± 8.1	0.064
**Legs (% fat)**				
All	32.9 ± 10.3	33.2± 11.1	34.5 ± 10.7	0.638
Women	41.7 ± 8.2	41.3 ± 6.6	40.8 ± 7.5	0.885
Men	27.2 ± 7.1	23.4 ± 6.4	24.9 ± 7.0	^b^ <0.05
**Trunk (% fat)**				
All	37.7 ± 8.5	33.8 ± 9.5	34.2 ± 9.6	^a^ <0.05
Women	41.0 ± 8.0	37.3 ± 8.3	35.5 ± 9.4	^a^ <0.05
Men	35.6 ± 8.3	29.5 ± 9.2	32.2 ± 9.7	^b^ <0.05
**Android (% fat)**				
All	43.0 ± 9.0	38.1 ± 10.4	38.6 ± 10.5	^a^ <0.05
Women	45.7 ± 8.3	41.3 ± 9.9	39.5 ± 9.9	^a^ <0.05
Men	41.2 ± 9.1	34.3 ± 9.8	37.3 ± 11.5	^b^ <0.05
**Gynoid (% fat)**				
All	39.6± 9.4	39.4 ± 10.3	40.4 ± 9.5	0.782
Women	47.4± 7.0	47.0 ± 5.7	46.3 ± 5.8	0.746
Men	34.6 ± 7.1	30.3 ± 6.5	31.6 ± 6.8	^a^ <0.05
**Total fat (%)**				
All	33.8 ± 8.5	31.9 ± 9.2	32.8 ± 9.0	0.383
Women	39.3 ± 7.4	37.3 ± 6.8	36.1 ± 8.1	0.197
Men	30.2 ± 7.3	25.4 ± 7.5	27.7 ± 8.1	^a^ 0.011
**Total fat mass (kg)**				
All	73.7 ± 12.1	67.6 ± 11.5	65.7 ± 11.4	^a^ <0.05
Women	65.8 ± 10.1	61.0 ± 8.4	60.3 ± 9.6	^a^ <0.05
Men	78.8 ± 10.5	75.6 ± 9.6	74.0 ± 8.8	0.100

Comparisons between groups

^a^ I versus II

^b^ I versus III; P values < 0.05 were considered significant. Abbreviations: % = percentage; SD = standard deviation.

**Fig 2:**
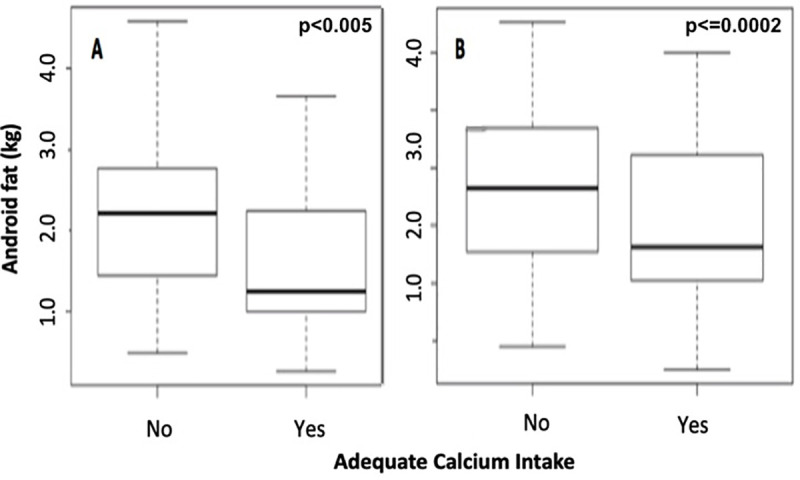
Android fat according to calcium intake in men (A) and women (B).

Android fat varied depending on calcium intake and was lower in men (1,583.34 ± 879.17 g versus 2,196.45 ± 917.91 g, p<0.005) and women (1,542.75 ± 768.14 g versus 1,828.45 ± 675.25 g, p = 0.0002) with normal calcium intake compared with those with inadequate intake, respectively (Fig 2).

Adequate calcium intake had a weak inverse correlation with BMI (r = -0.16, p = 0.007), WC (r = -0.18, p = 0.004), and android fat (r = -0. 25, p<0.001).

**Fig 3:**
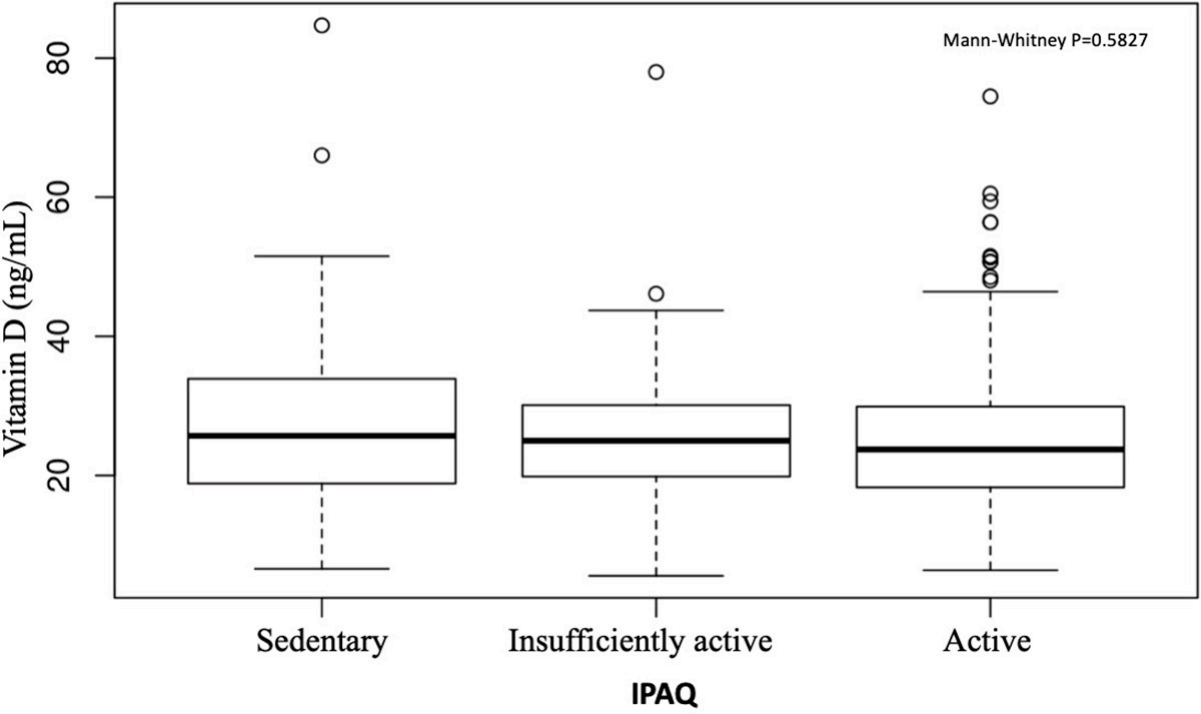
Vitamin D levels and physical activity.

The majority of the participants were considered active according to IPAQ results; specifically, 60 (20.06%) were sedentary, 77 (25.75%) were insufficiently active, and 162 (54.18%) were active. Physical activity levels interfered in body composition, with a higher percentage of trunk fat in sedentary compared with active individuals (p<0.001). Also, higher plasma glucose (>100 mg/dL) levels were found in 25.4% of the sedentary individuals compared with 4.8% of the active ones (p = 0.003), but 25OHD levels were comparable across groups with different physical activity levels (p = 0.5827), even when the groups were analyzed separately (p = 0.5731) (Fig 3).

The multivariate analysis was performed using as a dependent variable the 25OHD level and as independent variables age, gender, and all variables that emerged as significant in the univariate analysis (BMI, calcium intake, WC, IPAQ score, increased plasma glucose level, total fat [% and grams], trunk fat [% and grams], android fat, gynoid fat [%], arms fat [%]). The result of the multivariate analysis including all subjects showed that android fat (RR 0.44, 95% CI 0.24–0.80, p = 0.007) and WC (RR 1.01 CI, 95% CI 0.98–0.99, p < 0.001) maintained a negative effect on 25OHD level, while age had a positive influence on this parameter (RR 1.01, 95% CI 1.00–1.01, p < 0.001).

## Discussion

This study showed that in nonobese individuals, 25OHD levels are related to calcium intake, fat mass, fat distribution, and anthropometric parameters including BMI and WC. The groups divided according to vitamin D status were comparable in terms of age, race, comorbidities, and serum calcium and glucose levels. However, women had higher 25OHD levels than men, an observation that has already been reported in studies from Saudi Arabia and India [32, 33]. However, opposite findings have been reported by other studies [34, 35]; indeed, a study in older patients found no such difference between men and women [36]. The greater number of men in GI in the present study may be due to differences between sexes in terms of sun exposure and dietary diversity, or because of increased use of prophylactic vitamin D among women (which was not evaluated in this study). Sex differences in 25OHD levels is a controversial issue that deserves more studies and may be explained by variations in the presence of conditions or disorders that affect vitamin D metabolism, such as reduced or altered absorption, abnormal metabolism due to chronic kidney disease, or hepatic dysfunction [37], which were exclusion criteria for enrollment in the present study.

Values of BMI and WC were higher in individuals of both sexes with vitamin D deficiency and correlated inversely with 25OHD levels both in men and women. Other studies have also found that individuals who are overweight and obese have higher rates of vitamin D deficiency compared with those with normal weight [38, 39]. Similar to our study, Ardawi et al. found that serum 25OHD levels were lower in individuals in the upper quintiles of BMI and waist-to-hip ratio (WHR) [40]. This association can be explained by sequestration of vitamin D by visceral fat, resulting in large amounts of subcutaneous fat reducing circulating 25OHD [40]. In the VITAL study, baseline 25OHD levels varied according to age, sex, race, ethnicity, and BMI and were higher (33.6 ng/mL) in individuals with BMI < 25 kg/m^2^ and lower (27.8 ng/mL) in those with BMI ≥ 30 kg/m^2^ [41]. These findings have been confirmed in another study [42].

The body composition data in the present study showed an inverse relationship between 25OHD levels and different fat parameters in men and women. The negative effect of android fat and WC was confirmed by multivariate analysis. These findings are aligned with results reported by Al Hayek et al., who showed a higher percentage of body fat in individuals with low vitamin D levels compared with those with sufficient levels of this vitamin. Indeed, the authors reported an 8% increase in odds of 25OHD levels ≤ 30 ng/mL for each 1% increase in body fat after control for BMI and other confounders [43]. In contrast, other studies–including a meta-analysis of 10,898 individuals from three prospective European cohorts with longitudinal follow-up–found no correlation between vitamin D (25OHD) levels and fat mass [44–46].

Our study found no association between vitamin D (25OHD) levels and lean mass; this has also been reported in another study, which found that low vitamin D levels were associated with a higher percentage of body fat but not with lean mass [47]. Interestingly, the opposite has been observed in obese patients, in whom a high level of 25OHD was associated with greater lean body mass [48].

Adequate calcium intake correlated inversely with android fat in the present study and other studies, showing an association between low calcium intake and increased adiposity, especially in women [41]. These data are in agreement with findings from epidemiological studies that have shown an association between diet rich in calcium and weight loss [49] and loss of visceral fat independent of the restriction in energy consumption [50]. Observational studies have demonstrated that calcium intake is inversely associated with body weight [51]. However, controversial results emerged from a randomized controlled trial evaluating the effect of supplemental calcium with versus without vitamin D on weight management and metabolic profiles [52]. Other studies found no association of calcium intake with weight loss or body fat [53, 54], and reasonable explanations for the relationship between low calcium intake and fat accumulation are still lacking. Of note, calcium-sensing receptors may affect the accumulation of fat by mediating antilipolytic pathways in adipose tissue, as demonstrated in rats fed with low-calcium diets [55].

The present study found no correlation between 25OHD levels and physical activity, in contrast to findings by the D2d Research Group that showed that 25OHD levels are associated with nonwinter seasons, reduced WC, and increased physical activity [56]. However, the present study found a correlation between exercise levels and lower blood glucose levels with percentage of trunk fat in sedentary and active individuals.

Although levels of 25OHD correlated inversely with visceral fat, WC, and BMI in our study, they showed no correlation with blood glucose. The D2d trial analyzed whether vitamin D supplementation could lower the risk of type 2 diabetes and found no significant benefit of the vitamin in decreasing progression to diabetes in individuals with vitamin D sufficiency, although a *post hoc* analysis found a potential benefit of vitamin D supplementation in individuals with very low vitamin D levels [56]. The same was reported in a meta-analysis of the effectiveness of vitamin D supplementation in improving glycemic control in patients with type 2 diabetes who were nonobese and had vitamin D deficiency [57]. Corroborating the findings of the present study, Nikolova et al. found no association between fasting plasma glucose and 25OHD levels in women [12].

The present study has some limitations, including the lack of data on sun exposure and parathyroid hormone levels. Also, the focus of the study was not on a causality effect, but on the association of 25OHD levels with body fat distribution. Strengths of the study include the finding of new data in healthy, nonobese individuals in terms of body composition (mainly fat mass distribution) and 25OHD levels, which have only been reported before in patients with obesity and other diseases.

In conclusion, in healthy, nonobese individuals, low vitamin D levels were associated with a specific body composition pattern (greater BMI and WC, increased fat deposition in the trunk, android fat) that is commonly described as increasing the risk for metabolic syndrome.
